# The Effect of Marital Violence on Infertility Distress among A Sample of Turkish Women

**Published:** 2014-03-09

**Authors:** Aygül Akyüz, Gönül Şahiner, Memnun Seven, Bilal Bakır

**Affiliations:** 1 Department of Obstetrics and Gynecologic Nursing, Koc University School of Nursing, Istanbul, Turkey; 2 Department of Obstetrics and Gynecologic Nursing, Gulhane Military Medical Academy, School of Nursing, Ankara, Turkey; 3 Department of Public Health, Gulhane Military Medical Academy, School of Medicine, Ankara, Turkey

**Keywords:** Infertility, Violence, Distress

## Abstract

**Background:**

The aim of this study was to determine the relationship between marital violence and distress level among women with a diagnosis of infertility.

**Materials and Methods:**

This cross-sectional study consisted of 139 married women diagnosed as primary infertile who applied to an *in vitro* fertilization (IVF) center in Turkey, between September and December 2009. A descriptive information questionnaire developed by the researcher was used for data collection. In addition, an infertility distress scale (IDS) for determining the severity effect of infertility and the scale for marital violence against women (SDVW) for determining level of marital violence against the women were used.

**Results:**

The total IDS score of the study sample was 37.76 ± 10.53. There was no significant relationship between the age and education level of the women and the total IDS score. The total IDS score was higher in women who did not work and those being treated for infertility for more than three years. The total SDVW score of the study sample was 67.0 ± 8.26. The total SDVW score was higher in women who had been trying to have a child for more than six years and had received infertility treatment for longer than three years. The employment status of the women and physical, emotional, and sexual violence scores had a statistically significant relationship with the IDS scores. The emotional violence score was found to have the highest significance among the variables affecting total IDS score.

**Conclusion:**

Marital violence is a factor increasing the distress of infertile women. Healthcare staff serving infertile couples should consider the possibility of domestic violence against women as a factor affecting the psychological infertility distress level.

## Introduction

Infertility is defined as failure of pregnancy in a married couple despite appropriately timed intercourse (1). The inability of a married couple to become pregnant and to have children despite their desire to do so means they are unable to fully realize their objective of "becoming a family." Having children is a social responsibility for a family (2, 3). Inability to fulfill this responsibility adversely affects the social life, emotional status, marital relations, future plans, self-esteem, and body image of the couple. Infertility manifests itself as a sudden and unex- pected life crisis in which the diagnosis spreads over a long term and generates excessive stress while pushing adaptation mechanisms to the limit (4). A study by Cwikel et al. (5) found that approximately half of women undergoing fertility treatment rated infertility as the most stressful experience of their lives.

One methodologically unique study examined the psychological sequelae of infertility and the treatment failure among Chinese women. The prevalence of distress increased from 33 to 43% after treatment failure, while prevalence of depression remained constant (8%). The severity of depression following treatment failure was predicted by duration of infertility (6).

Couples live in fear and anxiety about infertility as well as the infertility diagnosis, treatment process, and treatment outcome (7). Each individual blames himself/herself and reflects his/ her anger to the other. This situation may cause conflict between the spouses, a decrease in selfesteem and in frequency of sexual intercourse, and the development of feelings of inadequacy in a female or a male. As a result, the bonds of marriage are put under psychological pressure: therefore, it can be a reason for marital incompatibility and also divorce (2, 8-10). The factors reducing marital harmony and satisfaction also cause domestic violence and are reported to increase the possibility of being subjected to domestic violence for women more than twofold (11).

In a study by Yıldızhan et al. (11), they found that 33.6% of women diagnosed with primary infertility had been subjected to domestic violence due to infertility. In this study, verbal abuse was the most common type of domestic violence reported (63.4%). The abused women (87%) had been threatened with divorce by their husbands. A study by Ardabily et al. (12) that determined the prevalence of and risk factors of domestic violence against women with female factor infertility indicated that 61.8% of women reported having experienced domestic violence because of their infertility.

The last guideline of world health organization (WHO) on international intervention aimed to alleviate the negative effects of infertility and to improve the quality of infertile couples’ lives using psychosocial intervention for both female and male (3, 13). The impact of infertility on the couples should be determined prior to psychosocial intervention. Violence is also reported to be a factor among the negative effects experienced by the couples (11). A literature survey on this subject reveals a limited number of studies. Only one study (14) investigates the distress caused by infertility on women, while no study was found showing the dimensions of the impact of violence on the relevant distress. It is important to know the relationship between infertility and violence as regards to planning the care that will be provided to couples receiving treatment for infertility and the use of ancillary procedures. Routine screening of infertile women in order to determine the probability of exposure to domestic violence and the early intervention when necessary are required to minimize the potential damage of violence.

Our study aimed to determine the effect of exposure to domestic violence on infertility distress in married women with an infertility diagnosis.

## Materials and Methods

### Participants

This cross-sectional study was carried out between September and December 2009 at the Infertility Center in Gulhane Military Medical Academy (GMMA) in Ankara, Turkey. Among 210 married women receiving treatment for primary infertility at this center during a-fourmonth data collection period, 152 women who met the criteria were asked to participate in the study using convenience sampling method. Of those selected, 144 (94.7%) consented to participate. After excluding 5 women due to incomplete data, the scope of the study consisted of 139 married women (91.4%).

### Measures

Data were obtained using the descriptive information questionnaire, infertility distress scale (IDS) (14), and scale for marital violence against women (SDVW) (15).

The IDS was developed by Akyuz et al. (14) in order to determine the level of the psychological effect caused by infertility and the treatment process in Turkish women. The scale included statements used to express emotional states. After reading each statement, the subject indicates how he/she feels in relation to not being able to have children. The IDS consists of 16 positive and five negative statements for a total of 21 items. Items 3, 10, 13, 14, and 21 are negative statements. Positive statements are scored between 1 (never) and 4 (always), while negative statements are scored in reverse. There are no subgroups of this scale; the lowest score is 21, while the highest is 84. A high score on the scale also means that the infertility distress level is high. The Cronbach’s alpha value of the scale was found to be 0.89 in our study.

The SDVW was developed by Kılıç (15) for the Turkish population. It includes 50 items in five subgroups. These subgroups include physical violence, emotional violence, verbal violence, economic violence, and sexual violence. Each group can be used separately. The total score indicates the level of marital violence against the women. The SDVW con- sists of positive and negative statements. Positive statements are scored between 1 (never), 2 (sometimes) and 3 (always), while negative statements are scored in reverse. Participants were asked to indicate the statement most appropriate to themselves. 

For example; 

(never) (sometimes) (always) "my husband insults me" 

() () ()

The minimum score is 50, while the maximum is 150. The scale has no cut-off point. The Cronbach’s alpha values obtained during the development of the scale ranged from 0.73 to 0.94. The Cronbach’s alpha coefficient was calculated as 0.83 in this study.

The descriptive information questionnaire was developed by the present investigators after an evaluation of the relevant literature. The validity of the content was examined by experts in the obstetrics field to confirm general appropriateness and applicability. The questionnaire consists of 21 questions and covers sociodemographic data, including the ages of the women and their spouses, level of education, occupational status, age at first marriage, and infertility characteristics. The prepared questionnaire was first administered to 20 infertile women at the *in vitro* fertilization (IVF) Unit of the hospital as a pilot study to ascertain whether the items could be easily understood.

### Procedure

 The Institutional Review Board of Gulhane Military Medical Academy approved this study. After the aim and method of the study were explained, the participating women provided verbal consent. Survey forms were filled out by the principal investigator through face-to-face interviews with each woman. The average time for an interview was approximately 25 minutes. 

### Data analysis

The SPSS 15.0 software package was used for statistical analysis. The distribution of the data was expressed as counts and percentages. The t test, Pearson correlation, Linear regression, and one-way ANOVA were used for statistical analyses, while a p value less than 0.05 was accepted as statistically significant. The backward method was chosen for the Linear regression analysis

## Results

Table 1 presents demographic information, duration of infertility and duration of treatment (Table 1). 

The mean IDS scores of the women participating in the study were 37.76 ± 10.53. There was no significant relationship between the age (t: 0.036, p=0.971) and education levels (F: 0.409, p=0.665) of the women and the total IDS score. The total IDS score was higher in women who did not work (t: 3.361, p=0.001) and those being treated for infertility for more than three years (t: 3.728, p<0.001). A spouse younger than 33 years (t: 2.115, p=0.036) with at least university-level education (t: 2.201, p=0.030) increased the total IDS score in women (Table 2).

**Table 1:** The socio-demographic features and infertility stories of the women and their partners


n=139	Χ ± SD			

**Age of women (Y)**	29.8 ± 4.99			
**Age of women’s partners (Y)**	33.5 ± 5.56			
**Duration of marriage (Y)**	6.98 ± 3.48			
	**Women’s partners n=13**		**Women n=139**	
	**n**	**%**	**n**	**%**
**Educational status**				
**Primary school**	33	23.7	-	-
**Secondary school**	76	54.7	65	46.7
**University or higher**	30	21.6	74	53.2
**Employment status**				
**Not working**	119	85.6	2	1.4
**Working**	20	14.4	137	98.6
**Type of work (n=20)**				
**Formal and regular**	14	70.0	120	87.5
**Informal**	6	30.0	17	12.5
**n=139**	Χ ± SD			
**Duration of infertility (Y)**	4.59 ± 3.36			
**Duration of infertility treatment (Y)**	3.14 ± 2.64			


There was no significant relationship between age (t: 1.046, p=0.298), education levels (F: 0.555, p=0.575), and employment status (t: 0.616, p=0.543) of the women and the total SDVW score in the study. The total SDVW score was higher in women who had been trying to have a child for more than six years (t: 2.432, p=0.016) and had received infertility treatment for longer than three years (t: 2.516, p=0.013). The SDVW mean scores of infertile women were 67.0 ± 8.26 (Table 3).

A weak positive correlation was found between the total violence, including emotional, verbal, economic, and sexual violence, scores that the women received from the SDVW scale and the total IDS score (p<0.05). There was no correlation between physical violence against women and their IDS score (Table 4).

Regression (linear) analysis was conducted in order to evaluate the relationship between the independent variables determined to affect infertility distress and the total IDS score. The employment status of the women and physical, emotional, and sexual violence scores were included in the model as it has a statistically significant relationship with the IDS scores. The value of the correlation between the total IDS score and the scores obtained from the model was 0.475. The Durbin-Watson coefficient had a value close to 2 (1.713), demonstrating that our model is well formed. The emotional violence score was found to have the highest significance among the variables affecting total IDS score (B: 0.329; p<0.001) (Table 5).

**Table 2:** Comparison of infertility status and socio-demographic characteristics of the women with the total IDS score.


n=139	Total IDS score		
	Χ ± SD	t/F*	P

**Age (Y)**			
**<30**	37.8 ± 8.23	0.036	0.971
**≥30**	37.7 ± 12.7		
*Education status			
**Primary school**	38.5 ± 12.9	0.409*	0.665
**Secondary school**	38.0 ± 11.0		
**University or higher**	36.2 ± 4.89		
**Employment status**			
**Not working **	38.5 ± 11.0	3.361	0.001
**Working**	33.3 ± 5.26		
**Duration of infertility treatment (Y)**			
**<3 years**	34.7 ± 7.14	3.728	<0.001
**≥3 years**	41.1 ± 12.6		
**Age of the women’s partners**			
**<33**	39.4 ± 11.7	2.115	0.036
**≥33**	35.7 ± 8.49		
**Partners’ education level**			
**High school or lower**	35.7 ± 7.83	2.201	0.030
**University or higher**	39.5 ± 12.2		
**The mean IDS score**	**Χ ± SD**		
	37.76 ± 10.53


*ANOVA

**Table 3:** Comparison of infertility status and socio-demographic characteristics of the women with the total SDVW score


n=139	Total IDS score		
	Χ ± SD	t/F*	P

**Age (Y)**			
**<30**	66.2 ± 5.74	1.046	0.298
**≥30**	67.8 ± 10.4		
*Education status			
**Primary school**	67.9 ± 8.22	0.555*	0.575
**Secondary school**	67.1 ± 6.77		
**University or higher**	65.7 ± 11.3		
**Employment status**			
**Not working **	67.1 ± 8.36	0.616	0.543
**Working**	66.0 ± 7.75		
**Duration of infertility (Y)**			
**<6 years**	66.0 ± 6.90	2.432	0.016
**≥6 years**	69.8 ± 10.9		
**Duration of infertility treatment (Y)**			
**<3 years**	65.3 ± 6.78	2.516	0.013
**≥3 years**	68.8 ± 9.38		
**The mean IDS score**	**Χ ± SD**		
	67.0 ± 8.26


*ANOVA

**Table 4:** Comparison of total IDS score and the SDVW score


**Violence subgroups of SDVW**	Total IDS score	
	P	r

**Total SDVW score**	0.289	0.001
**Physical violence**	0.038	0.661
**Emotional violence**	0.360	0.001
**Verbal violence**	0.267	0.001
**Economic violence**	0.182	0.032
**Sexual violence**	0.263	0.002


**Table 5:** The results of regression analysis between the independent variables affecting infertility distress and the total IDS score


	Total IDS score		
	B	T	P

**Independent variables determined to affect total IDS score**			
**Total physical violence score**	0.344	-3.180	0.002
**Total emotional violence score**	0.329	3.642	<0.001
**Total sexual violence score**	0.334	2.892	0.004
**Employment status of the women**	0.183	-2.410	0.017
****			
	**R**	**R square**	**Durbin-Watson**
**IDS model**	0.475	0.225	1.713


## Discussion

Data related to the infertility distress level, the status of being subjected to violence, and the effect of the violence on the infertility distress level of infertile women are discussed in this section. This is the first study showing the effect of violence against infertile women on the infertility distress level. There are very few studies investigating the special status of violence against infertile women, and there is no study showing the effect of violence on the distress caused by infertility in the literature.

The mean IDS score of the women in this study was 37.76 ± 10.53. The IDS mean score of the women was determined as 45.94 ± 10.9 in the validation studies of the scale by Akyuz et al. (14), Ünal et al. (16), in which they both found the IDS mean score of the women to be 39.01 ± 9.6. The moderate scale scores of women in these studies indicate that women are emotionally affected to a moderate degree by their inability to have children.

No significant relationship was found between the age and education levels of the women and the infertility distress level in our study. Ünal et al. (16) determined that the infertility distress level increased with age and decreased with increasthought to arise from other factors contributing to the infertility distress level. The infertility distress level was lower in employed women. Working may generate a social environment that facilitates coping in women with infertility and supports the women (17). Working at an income-generating job, being productive, and being recognized in the community also compensate to a certain degree for the effect of losing a fertility-related role (18). The infertility distress level of women also increased with increased duration of infertility treatment. These results are parallel to other results from the literature (14, 16, 19, 20). An increase in the duration of infertility is thought to cause an increase in stress by gradually decreasing women’s hopes to have children.

Infertility is also thought to be a factor causing domestic violence. Infertile women are twice as likely to be subjected to domestic violence than other women (11). Ardabily et al. (12) similarly found that 61.8% of women were subjected to domestic violence due to infertility. The SDVW mean scores of infertile women was 67.0 ± 8.26 in this study. According to the nationwide survey about violence against women in Turkey, 35% of women have experienced physical violence from their husbands at least once in their lives (21). Similar studies in the literature on the subject indicated high rates of domestic violence against infertile women (11, 22, 23). Yıldızhan et al. (11) stated that 78% of infertile women were subjected to violence for the first time after their infertility diagnosis.

There was no correlation between the age, education level, and occupational status of the women and the status of being subjected to violence in this study. A few studies in the literature on similar subjects also found no significant association between age and violence among infertile women subjected to violence (11, 12). The lack of a significant relationship between age, education level, and occupational status and violence in infertile women in our study and other studies is an important finding that shows violence is experienced widely in all areas of human life, regardless of economic develop- ment and education level. Results from the study of the Women’s Solidarity Foundation (2007) indicated that 438 women who visited the solidarity foundation had varying education levels, incomes, and ages (24). This result demonstrates that age, education level and income status did not protect women from violence. These findings support our study results and interpretation. Women receiving infertility treatment for an extended time and waiting to have children are subjected to an increased amount of violence. This situation is thought to develop in connection with living under stress for a longer duration and decreased marital harmony of the couple.

Violence was found to increase the infertility distress level in this study. Violence can be experienced in physical, economic, emotional, and verbal areas (25, 26). Infertile women who are exposed to emotional and sexual violence demonstrate a higher infertility distress level. There is no study showing the infertility distress level in women subjected to violence. However, rates of sexual and emotional violence are stated to be higher in studies of infertile women than in population-based studies (10, 11, 12, 22, 23, 27). This situation is thought to arise from the emotional influence experienced during the diagnosis and treatment process. Many studies have also indicated that marital relations of couples become worse and sexual satisfaction is decreased, especially with repeated unsuccessful attempts (2, 9, 28, 29). Infertile women subjected to physical violence have a significantly higher infertility distress level, but their emotional and sexual violence scores are lower. Physical violence is the type of violence that hurts and harms women, and is likely to result in physical damage. Injury and trauma caused by physical violence negatively affects marital relations and the selfimage of women (25). Infertile women may push their wish of having a child into the background because of the physical violence they are subjected to. In other words, women subjected to physical violence may show less inclination to undergo infertility treatment. On the other hand, women with high emotional scores may have presented themselves as being subjected to violence because of their own perception, although the reality is different. A woman may experience anxiety and feels guilty of being infertile and imperfect. 

## Conclusion

These results are important as they show that infertile women may be at risk for violence, demonstrate that the women’s psychological infertility distress level, and show that the effect of domestic violence on infertility distress in women. Compliance to treatment and success of therapy are known to be changed due to the psychological influence of infertility on the woman. In this context, healthcare staff serving infertile couples should consider the possibility of domestic violence against women as a factor that affects the psychological infertility distress level. Infertile couples thought to be at risk should, therefore, be followed more closely.

The sample of this study is made up of a specific group of Turkish women. The study was also conducted at a single center. It should, therefore, be noted that these results reflect only a group of Turkish women receiving infertility treatment and that the socio-cultural differences may affect both violence and infertility data. The status of being subjected to domestic violence in women receiving infertility treatment and the effect of violence on infertility distress should, therefore, be assessed in communities with different socio-cultural characteristics. 

This study evaluated the status of being subjected to domestic violence in women receiving infertility treatment and the effect of violence on infertility distress with a quantitative method. Therefore, both qualitative and quantitative research is recommended to reveal the exposure to violence in women receiving infertility treatment.
